# Editorial: Individual differences in second/foreign language speech production: multidisciplinary approaches and new sounds

**DOI:** 10.3389/fpsyg.2023.1224059

**Published:** 2023-06-13

**Authors:** Peijian Paul Sun, Boping Yuan, Xun Yan, Jimin Kahng

**Affiliations:** ^1^Department of Linguistics, Zhejiang University, Hangzhou, China; ^2^School of Foreign Languages, Shanghai Jiao Tong University, Shanghai, China; ^3^Faculty of Asian and Middle Eastern Studies, University of Cambridge, Cambridge, United Kingdom; ^4^Department of Linguistics, University of Illinois at Urbana-Champaign, Champaign, IL, United States; ^5^Department of Modern Languages, University of Mississippi, Oxford, MS, United States

**Keywords:** individual difference, L2 speech production, fluency, test performance, gesture

Second/foreign language (L2) speech production can be subject to various individual difference factors. A review of the literature indicates that common individual difference factors examined in the field of L2 speaking include, but are not limited to, learning environments, learning strategies, willingness-to-communicate, motivation, and anxiety (Sun and Teng, 2021; Sun, 2022, 2023; Sun and Zhang, 2022). Although there is no consensus on what individual differences encompass, the literature has witnessed a growing interest in research on individual differences in L2 speaking. Nevertheless, our understanding of L2 speaking is still in its infancy compared to L2 writing. We, therefore, call for more research to shed light on the possible influence of individual difference factors on L2 speaking from multidisciplinary approaches.

Our call has resulted in 10 seminal works on individual differences in L2 speech performance and production (9 original research, 1 brief report, and 1 opinion). Specifically, three papers examined individual differences in L2 fluency (Aubrey; Feng; Kahng), two profiled L2 English speakers' oral test performance (Gao; Zhang et al.), and two tapped into elicited imitation in L2 speech production (Lei and Yan; Munro). The rest three papers explored the role of first language (L1, Zhang and Yuan) and gesture (Ma and Jin) in L2 speech production, and the connectedness in L2 speech development (Botezatu et al.).

## Individual differences and L2 fluency

To understand the relationship between emotions (i.e., anxiety and enjoyment) and breakdown fluency from an intra-individual level, Aubrey adopted an idiodynamic approach to investigate English-as-a-foreign-language (EFL) university students' L2 speech performance in monolog tasks. The study found a strong positive connection between anxiety and breakdown fluency, but a weak association between enjoyment and breakdown fluency. The study also suggested that task design, task implementation, cognitive-linguistic, and achievement outcome factors could influence the emotion-fluency relationship.

To understand the L2 fluency development from a longitudinal perspective, Kahng examined L1 Chinese EFL learners' L2 utterance fluency change (i.e., speech, pausing, and repair) over 5-month study-abroad. The study showed that there was an improvement in learners' mean syllable duration and end-clause silent pausing. Such a change was the result of learners' increased L2 use rather than their L2 motivation.

However, both Scott's and Kahng's studies focused on within-individuals' rather than between-individuals' L2 fluency. In other words, L2 fluency should be examined not only from an individual perspective but also from a dialogic perspective to take into account the interaction between interlocutors. As Feng pointed out, incorporating both the monadic (intra-individual) and non-monadic (inter-individual) perspectives could enrich the ongoing discussion on factors contributing to L2 speech performance.

## Test takers' L2 speech performance

Do high-proficiency EFL speakers differ in oral test performance? If yes, how can we create profiles to capture these differences? Gao's study has provided the audience with detailed steps for profiling high-proficiency EFL speakers' L2 speech performance. Specifically, employing cluster analysis on participants' speech fluency and vocabulary use, the study identified four types of high-proficiency EFL speakers.

In addition to Gao's approach to profiling high-proficiency EFL speakers' oral test performance, Zhang et al. examined Chinese EFL learners' speaking performance in integrated L2 speaking assessment tasks. The study revealed that problem-solving was the most frequently adopted metacognitive strategy in L2 speech production. However, metacognitive strategies were not found to have significant effects on participants' L2 speech performance across the four integrated speaking tasks in the study.

Regardless of test-takers' different profiles of L2 speech performance or different use of metacognitive strategies in L2 speech production, it is necessary to take test contexts into consideration. For example, to what extent can test anxiety and time pressure influence test takers' L2 speech performance? How different learners' L2 speech performance profiles or speaking strategies use will be in testing and non-testing conditions? Future research may consider addressing these issues.

## Elicited imitation and L2 speech production

Elicited imitation (EI) is an effective measure of L2 proficiency (Yan et al., 2016, 2020). It requires participants to listen to a series of stimulus sentences, phrases, words, or sounds and then repeat them verbatim (Underhill, 1987). Despite the surge of interest in EI, little is known about the strategies employed by L2 learners in EI tasks and their effect on EI performance. Lei and Yun bridged the gap by examining L2 Chinese learners' strategy use in EI tasks. They found that participants adopted cognitive, metacognitive, communication, approach, and test-wiseness strategies in the process of elicited imitation. Additionally, cognitive strategies were found to have a significant positive influence on EI prompted L2 speech performance, while metacognitive strategies had a significant negative effect.

While differences in strategy use can impact EI performance in L2 speaking, the extent to which differences in ET tasks may influence learners' L2 speech performance in vowel production is under-researched. Munro, therefore, examined L1 Cantonese speakers' production of English high vowels (i.e.,/i/,/ı/,/u/,/℧/) on two EI tasks: a picture naming task and an interrupted repetition task. The study found that participants' vowel production on the interrupted repetition task was more intelligible than on the picture naming task by over 10 percentage points. However, the task effect on vowel production was inconsistent across speakers.

## L1, gesture, and graph structure in L2 speech production

This collection also attracted researchers to explore L2 speech production from the angles of L1, gesture, and graph structure. Specifically, Zhang and Yuan investigated the influence of learners' L1 English and L1 Korean on their L2 Chinese oral production of ellipses. The study revealed that L1 influence on L2 Chinese learners' oral production of ellipses was not observed at the beginner level but observed at the intermediate and advanced levels. Ma and Jin investigated the relationship between co-speech gestures and L2 speech performance. Results showed that there were positive correlations between co-speech gestures and L2 speech measures in meaning and discourse. Last but not least, Botezatu et al.'s graph structure analysis of the relationship between L2 lexical retrieval and the global connectedness of narratives suggested that, in the early phases of L2 oral development, the connectedness of L2 speech can be attributed to individuals' ability in L2 lexical access.

Summing up, this collection of articles provides the audience with a multidisciplinary approach to understanding how individual difference factors may be associated with L2 speech production. The studies in the collection offer valuable insights into the complex nature of L2 speech production and highlight the importance of taking individual differences into account to support learners' L2 speech development.

## Author contributions

PS drafted the editorial. All authors contributed to the refinement of the editorial and approved it for publication.
